# Retinal vein occlusions associated or complicated with glaucoma. Aspects of prediction and paths of progression

**DOI:** 10.22336/rjo.2023.18

**Published:** 2023

**Authors:** Diana-Maria Dărăbuș, Cristina-Patricia Pac, Mihnea Munteanu

**Affiliations:** * Department of Ophthalmology, “Victor Babeş” University of Medicine and Pharmacy, Timişoara, Romania

**Keywords:** open angle glaucoma, retinal vein occlusions, neovascular glaucoma, intraocular pressure, cup-disc ratio, retinal nerve fiber layer

## Abstract

**Background and Objectives:** The aim of the study is to evaluate prediction factors and progression paths when retinal vein occlusions are associated with preexisting glaucoma or complicated with neovascular glaucoma.

**Materials and Methods:** The study included 111 patients diagnosed with retinal vein occlusions, of whom 21 with preexisting open angle glaucoma and 12 with neovascular glaucoma as complication. The study was conducted from September 2020 to September 2022 in Timişoara, Romania. We assessed intraocular pressure, cup-disc ratio and retinal nerve fiber layer from the moment of retinal vein occlusion diagnosis until at least one year of follow-up, considering these aspects as values of prediction concerning the paths of progression when glaucoma and retinal vein occlusions come together.

**Results:** The mean initial IOP for the affected eyes was higher (15.89 ± 2.73) than for fellow eyes (15.20 ± 3.11), with an increase of the IOP after one year, but with no statistically significant differences for the affected eyes (p=0.116) or for the other eyes (p=0.684), neither for the affected eyes associated with glaucoma in comparison with affected eyes without glaucoma association. The mean cup-disc ratio was higher for the affected eyes in comparison with the fellow eyes (0.4812 ± 0.219 for the affected eyes and 0.4738 ± 0.229 for the fellow ones in cases without associated glaucoma and 0.681 ± 0.157 for the affected eyes and 0.600 ± 0.241 for the fellow eyes in cases with associated glaucoma), with statistical significant differences in the evolution for both groups in comparison with the unaffected eyes (p=0.0056 for the first group and p=0.0003 for the second group). Comparing the evolution of the affected eyes with the preexisting glaucoma and the affected eyes without preexisting glaucoma, no statistical difference has been found (p=0.1104). The mean retinal nerve fiber layer decreased significantly in affected eyes without glaucoma (from 96 ± 14.71 to 89.16 ± 13.07) and in affected eyes with associated glaucoma (from 78.50 ± 4.23 to 75.50 ± 5.83), but with no significant differences (p=0.182). The level of decreasing was significantly more consistent in association with a venous occlusion (p= 0.0001).

**Conclusions:** The findings of the current study fortify the correlation between glaucoma as a risk factor for retinal venous occlusion development, the intraocular pressure and optic nerve cupping as prediction factors in retinal venous occlusions, the association of a well-controlled preexisting glaucoma with no effect on the progression of the retinal venous occlusions and the development of a neovascular glaucoma with a much aggressive and different path of disease progression.

## Introduction

Retinal vein occlusions represent one of the most common retinal vascular pathologies [**1**]. Several correlations between RVO and other pathologies have been established, some of them very well demonstrated over the years, but others still in need for closer scientific approach [**2**,**3**]. Glaucoma is considered one of those pathologies still in need for a closer look regarding the association with retinal vein occlusions. Although it has some common pathogenetic theories with the occurrence of retinal vein occlusions, they seem to be valid only for a small category of patients [**4**]. Several studies already demonstrated that glaucoma represents a higher risk for developing retinal vein occlusions [**5**,**6**], and the reasons seem to be the modifications produced at the lamina cribrosa, which consequently have an impact on the path of the vein passing through the optic nerve head [**4**]. Nevertheless, opinions and results vary when discussing the morphological and functional impact of glaucoma association with retinal vein occlusions. Considering intraocular pressure, retinal venous occlusions associated with high intraocular pressures with a subsequent fall of the IOP right after the occlusion have been reported [**7**]. Still, some authors report no difference concerning the incidence of the venous occlusions between patients with or without raised intraocular pressure, under similar medical conditions, questioning the role of intraocular pressure in the etiology of RVO [**3**]. A higher cup-disc ratio is associated with retinal vein occlusions [**8**], considered to be a prediction factor and retinal nerve fiber layer thickness a matter of progression. Therefore, we undertook this study to evaluate these initial morphological and functional aspects and the same aspects in evolution, for the affected eyes, in comparison with the unaffected ones. Every aspect that we chose to observe was analyzed from the perspective of an associated glaucoma or not.

## Materials and Methods

*Study Design and Population*

The present study was conducted from September 2020 to September 2022. 111 eyes of 111 patients diagnosed with unilateral central retinal vein occlusion or branch retinal vein occlusion were included in the study, of which 21 patients were previously diagnosed with open angle glaucoma and 12 others developed neovascular glaucoma as a complication. All patients signed an informed consent according to the institutional guidelines. The study is part of a doctoral dissertation that was approved by the Ethics Committee of “Victor Babeş” University of Medicine and Pharmacy Timişoara (no. 68/ 2020). The intraocular pressure variations, structural changes (retinal nerve fiber layer, cup disc ratio) were analyzed comparatively with the congener eye and with the other patients with retinal vein occlusions, but not in correlation with glaucoma. The follow up period was 12 months. Patients included in the study have been split into two groups, one group with associated glaucoma and another one without associated glaucoma. Taking into consideration the intraocular pressure (IOP), cup/ disc ratio and retinal nerve fiber layer, we compared the initial and after one-year measurements and also the evolution of these parameters between the two groups of patients, but also between the affected eyes (no matter if in association with glaucoma or not) and the fellow eyes.

*Data Collection*

The inclusion criteria were patients with branch retinal vein occlusion or central retinal vein occlusion, with BCVA more than 0.02, with (H=1) or without (H=0) associated open angle glaucoma already diagnosed before the venous occlusion, with (M=1) or without (M=0) neovascular glaucoma as a complication of the venous occlusion. Patients included in the study were those who were registered in the study center and completed all required follow-ups, investigations and treatments during at least one year of observation. The exclusion criteria were patients with BCVA less than 0.02, eyes with optic nerve swelling only when assessing RNFL due to an unreal associated RNFL value, non-compliant patients with treatment, follow-ups or investigations, mixed occlusions, associated diabetes (due to possible diabetic neuropathy or neovascular implication), associated unoperated cataract. The following data were collected: visual acuity at the beginning for inclusion/ exclusion criteria, approximate time of occlusion, age, preexisting open angle glaucoma and number of drops, intraocular pressure using Goldman applanation tonometry with adjustment depending on corneal central thickness, at first and during every follow-up. The IOP adjustment regarding corneal thickness was made using Schwind Sirius Camera, glaucoma analysis mode, by manually introducing the intraocular pressure value after applanation tonometry with the automatic adjustment using Dressner formula. Optic disc morphology using optic coherence tomography (Cirrus 5000, Zeiss) at first and during every follow-up and slit lamp examination for the anterior pole and dilated pupil fundus examination (DFE). C/ D ratio and retinal nerve fiber layer have been assessed, using optic disc cube 200x200 report from Cirrus and DFE for optic disc swelling as an exclusion criterion when analyzing retinal nerve fiber layer.

*Statistical Analysis*

Statistical calculations were performed using SPSS (Version 20, IBM), Microsoft Excel (2016, Microsoft Corporation, Redmond, WA, USA). For the variables of concern, elements of descriptive statistics have been computed (minimal value, maximal value, mean, standard deviation), and statistical tests have been applied to determine the significant differences (t-test). The graphical representations that have been used are the Box-Plot diagram and the error bar diagram (mean and 95% confidence interval for mean).

## Results

*Glaucoma as a risk factor*

During the selected period of time for the study, 32.735 patients have been registered in our Department and among these patients, 873 have been diagnosed with open angle glaucoma, which means 2,66%. This percent was significantly lower than preexisting open angle glaucoma between patients diagnosed with venous retinal occlusions in the Department and during the same period of time, more exactly 18.91% (21 cases out of 111).

*Intraocular pressure*

**Fig. 1:**
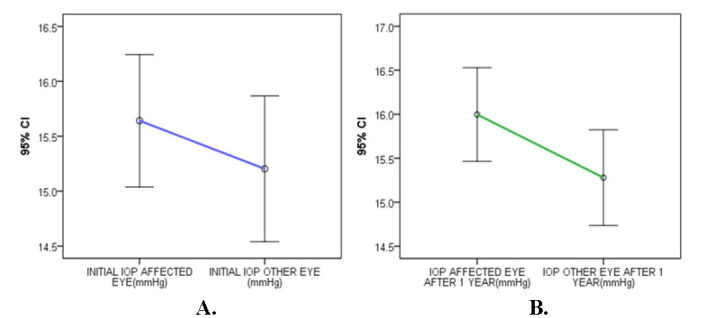
**A.** Comparison of the initial mean intraocular pressure (IOP) between affected and non-affected eye; **B.** Comparison of the mean intraocular pressure (IOP) between affected and non-affected eye after 1 year

For this analysis, patients who developed neovascular glaucoma (M=1) were excluded due to high intraocular pressures associated with this complication. The mean initial IOP for the affected eyes was higher (15.89 ± 2.73) than the fellow eyes (15.20 ± 3.11) with an increase of the IOP after one year, when affected eyes showed a mean IOP of 16.15 ± 2.40 and fellow eyes a mean IOP of 15.27 ± 2.55 (**Fig. 1**). After one year of monitorization, we did not register statistically significant differences concerning the IOP for the affected eyes (t=-1.587, p=0.116>0.05) or for the other eyes (t=0.409, p=0.684) (**Table 1**). The IOP increase during the first year was not statistically significant between eyes with venous occlusion and unaffected ones (t=0.764, P =0.4458 >0.05).

**Table 1:** IOP evolution after 1 year, affected eyes and congener ones (abbreviation: IOP = intraocular pressure; STD = standard deviation)

	Paired Differences						
			95% Confidence Interval of the Difference				
	Mean	STD	Lower	Upper	T	Df	Sig. (2-tailed)
Initial IOP affected eye (mmHg)-IOP affected eye after 1 year (mmHg)	-0.26667	1.67155	-0.60005	0.06672	-1.587	98	0.116
Initial IOP other eye (mmHg) - IOP other eye after 1 year (mmHg)	-0.07586	1.73017	-0.44461	0.29289	-0.409	86	0.684

Also, the evolution of the IOP in the affected eye was not statistically different in eyes with associated glaucoma (without changing the initial treatment) and those without associated glaucoma (**Table 2**).

**Table 2:** IOP evolution affected eyes without (H=0) and with associated glaucoma (H=1)

			95% Confidence Interval of the Difference			
Initial IOP affected eye (mmHg) - IOP affected eye after 1 year (mmHg)	Mean	Lower	Upper	T	Df	Sig. (2-tailed)
H=0	-0.33846	-0.73547	0.05855	-1.698	77	0.094
H=1	0.00	-0.5863	0.5863	0.00	20	1.000

*Cup-disc (C/D) ratio*

When analyzing the cup/ disc ratio, patients were split into two groups, those with associated open angle glaucoma, but not neovascular glaucoma (H=1 and M=0) and those without associated glaucoma (H=0 and M=0). The initial cup/ disc ratio was compared between affected eyes and fellow ones, separately for each group, and a higher cup/ disc ratio was registered in the affected eyes. For the first group of patients (M=0, H=0), the mean C/ D ratio was of 0.4812 ± 0.219 for the affected eyes and 0.4738 ± 0.229 for the fellow ones (**Table 3**). After 1 year, statistically significant differences have been registered regarding the cup/ disc ratio both for the affected eye and for the other one (**Table 4**). Also, judging from the evolutive perspective, the difference in the way this morphological aspect evolved in one year, it was statistically significant between the affected eye and the unaffected ones (t=2.812, p=0.0056<0.05).

**Table 3:** Cup-disc ratio mean and standard deviation for affected eyes and unaffected ones, without associated glaucoma (H=0, M=0) (abbreviation: C/ D ratio = cup-disc ratio; STD = standard deviation)

	Mean	N	STD	Std. Error Mean
Initial C/ D ratio affected eye	0.4812	78	0.21913	0.02481
C/ D ratio affected eye after 1 year	0.5181	78	0.21893	0.02479
Initial C/D ratio other eye	0.4738	78	0.22994	0.02604
C/ D ratio other eye after 1 year	0.4865	78	0.22288	0.02524

**Table 4:** Cup-disc ratio from an evolutive perspective for affected eyes and unaffected ones, without associated glaucoma (H=0, M=0) (abbreviation: C/ D ratio = cup-disc ratio; STD = standard deviation)

	Paired Differences						
			95% Confidence Interval of the Difference				
	Mean	STD	Lower	Upper	T	Df	Sig. (2-tailed)
Initial C/ D ratio affected eye - C/ D ratio affected eye after 1 year	-0.03692	0.06796	-0.05225	-0.02160	-4.798	77	0.000
Initial C/ D ratio other eye - C/ D ratio other eye after 1 year	-0.01269	0.03425	-0.02041	-0.00497	-3.273	77	0.002

For the second group of patients (M=0, H=1), the mean C/ D ratio was of 0.681 ± 0.157 for the affected eyes and 0.600 ± 0.241 for the unaffected ones (**Table 5**). After 1 year, statistically significant differences have been registered regarding the cup/ disc ratio both for the affected eye and for the other one (**Table 6**). Also, judging from the evolutive perspective, the difference in the way this morphological aspect evolved in one year, it was statistically significant between the affected eye and the fellow eyes (t=3.974, p=0.0003<0.05).

**Table 5:** Cup-disc ratio mean and standard deviation for affected eyes and unaffected ones, with associated preexisting glaucoma (H=1, M=0) (abbreviation: C/ D ratio = cup-disc ratio; STD = standard deviation)

	Mean	N	STD	Std. Error Mean
Initial C/ D ratio affected eye	0.6814	21	0.15784	0.03444
C/ D ratio affected eye after 1 year	0.6943	21	0.15148	0.03306
Initial C/D ratio other eye	0.6000	21	0.24199	0.05281
C/ D ratio other eye after 1 year	0.6029	21	0.23829	0.05200

**Table 6:** Cup-disc ratio from an evolutive perspective, for affected eyes and unaffected ones, with associated glaucoma (H=1, M=0) (abbreviation: C/ D ratio = cup-disc ratio; STD = standard deviation)

	Paired Differences						
			95% Confidence Interval of the Difference				
	Mean	STD	Lower	Upper	T	Df	Sig. (2-tailed)
Initial C/ D ratio affected eye - C/ D ratio affected eye after 1 year	-0.01286	0.01056	-0.01766	-0.02160	-0.00805	20	0.000
Initial C/ D ratio other eye - C/ D ratio other eye after 1 year	-0.00286	0.00463	-0.00496	-0.00075	-2.828	20	0.010

**Fig. 2:**
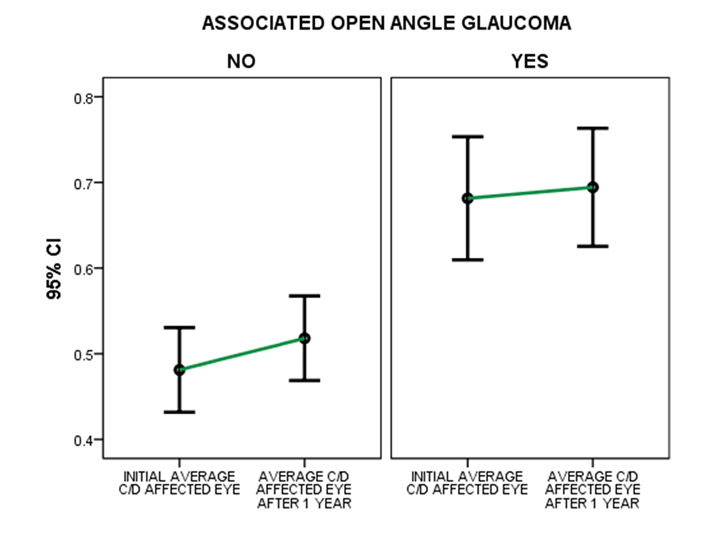
**Left:** Comparison of the evolution of the mean cup-disc ratio for the affected eye with no pre-existing glaucoma; **Right:** Comparison of the evolution of the mean cup-disc ratio for the affected eye with pre-existing glaucoma

Taking into consideration the evolution of this ratio in one-year time, no statistically significant differences have been found between affected eyes with associated glaucoma and affected eyes with no associated glaucoma (t=1.611, p=0.1104>0.05) (**Fig. 2**).

*Retinal nerve fiber layer*

Peripapillary retinal nerve fiber layer has been taken into consideration at first, comparing the affected eyes by being split into two groups, one without associated glaucoma (H=0, M=0) and the other with associated glaucoma (H=1, M=0). The mean retinal nerve fiber layer decreased from 96 ± 14.71 to 89.16 ± 13.07 after one year for the first group and from 78.50 ± 4.23 to 75.50 ± 5.83 for the second group. Both groups registered a statistically significant decrease of the retinal nerve fiber layer after one year (**Table 7**), but with no statistically significant differences between the two groups (t=-1.332, p=0.182 >0.05).

**Table 7:** Peripapillary retinal nerve fiber layer (pRNFL), from an evolutive perspective, in comparison, for affected eyes without associated glaucoma and affected eyes with associated glaucoma (abbreviation: STD = standard deviation; H=O = no preexisting glaucoma; H=1 = preexisting glaucoma; M=0 = no neovascular glaucoma)

	Paired Differences						
			95% Confidence Interval of the Difference				
Initial RNFL affected eye-RNFL affected eye after 1 year	Mean	STD	Lower	Upper	T	Df	Sig. (2-tailed)
H=0 M=0	6.83333	12.08578	3.53455	10.13212	4.155	53	0.0000
H=1 M=0	3	2.22288	1.89459	4.10541	5.726	17	0.0000

Secondly, all cases, except for those with neovascular glaucoma and optic nerve swelling, have been taken into consideration for the comparison of the retinal nerve fiber layer in dynamics for the affected eyes and the non-affected ones. Initially, higher values have been registered for the affected eyes group (mean values of 91.62 ± 14.97) than for non-affected eyes (mean values of 85.44 ± 14.23). After one year, the mean values for the first group were 85.75 ± 12.99 and 84.34 ± 14.04 for the second group. Therefore, in evolution, statistically significant differences between the two groups have been registered (t=3.962, p=0.0001<0.05), the level of decreasing being much more consistent in association with a venous occlusion. 

*Neovascular glaucoma*

All 12 cases of neovascular glaucoma reported as a complication were associated with ischemic central retinal vein occlusion, with a very low visual acuity (less than 0.05) from the first moment the patients started being ophthalmologically supervised. None of these cases had preexisting glaucoma and all the 12 cases initially had moderate cupping (C/ D ratio between 0.5 and 0.8) and severe cupping (C/ D ratio more than 0.8) after one year.

## Discussion

Considering that the frequency of glaucoma cases, in our study, was much higher among patients with retinal venous occlusions (18.91%) than among the general population (2.66%), glaucoma could be regarded as a risk factor for retinal vein occlusions. This result certifies what Yin X et al. underlined after a meta-analysis of previous studies, that we should always take glaucoma into consideration when analyzing patients with retinal vein occlusions [**2**].

Our study showed that a higher intraocular pressure, although in normal limits, could be a trigger for venous occlusions, associated with reduced blood flow or poor vasculature. We registered statistically significant higher intraocular pressures in the affected eyes in comparison with the non-affected ones, similarly with the study of Frucht J et al. [**9**]. Still, the evolution of the IOP in one year was not statistically different between the two groups and neither between the eyes with associated open angle glaucoma and those without associated open angle glaucoma. Therefore, the role of the intraocular pressure and the potential necessity of a hypotensive treatment for the fellow eye, as a prevention, is still worthy to be considered. Previous studies come into contradiction regarding the importance of the C/ D ratio in retinal vein occlusion development, some stating that eyes with large cup to disc ratios are at greater risk of having or developing an RVO than those with smaller ratios [**10**], meanwhile others state that there is no importance of the optic disc cup for the occurrence of retinal vein occlusions [**11**].

Our study found that the mean C/ D ratio was larger in cases in which the venous occlusion occurred than the mean C/ D ratio of the fellow eyes, underlying the fact that the risk is real, although the exact mechanism still cannot be explained. After one year, there have not been statistically significant differences between the morphological changes registered in the affected eyes with associated glaucoma and affected eyes without associated glaucoma, fact that raises the assumption that an associated glaucoma, with all its optic nerve perfusion theories, will not influence the morphological changes of the optic nerve in eyes with venous occlusions. Nevertheless, statistically significant differences have been noted when comparing the evolution between affected eyes with associated glaucoma and fellow eyes only with glaucoma underlying the effect that venous occlusions have on the optic nerve stability. 

Our result showed that peripapillary retinal nerve fiber layer (pRNFL) will most probably decrease after venous occlusions, showing an optic nerve damage, taking into consideration the fact that affected eyes presented a significant decrease in evolution of the pRNFL in comparison with the unaffected eyes. Similar results have been described by Ahn J et al. in 2021 [**12**]. Some authors believe that retinal nerve fiber layer thinning should be considered the “natural course after branch retinal vein occlusions” [**13**], meanwhile others tried to underline that “optic disc swelling can cause difficulties in evaluating ONH parameters” [**14**]. Moreover, our study showed that no statistical differences between affected eyes with preexisting glaucoma and affected eyes without preexisting glaucoma have been found, which demonstrates that an associated glaucoma with a proper control of the intraocular pressure will not accelerate or have a direct contribution on the nerve fiber layer thinning throughout the years. In comparison with the study of Chen HF et al. regarding neovascular glaucoma [**15**], we also agreed that this type of glaucoma develops as a complication after an ischemic type of occlusion in 100% of the cases, also with the fact that is highly associated with moderate to severe cupping in most of the cases, but contrary to what their study underlined, all our neovascular glaucoma cases developed without association with a preexisting open angle glaucoma.

There are several limitations to our study. One of them is the impossibility of evaluating each patient in the same period of time from the moment of the occlusion, because of very different moments chosen by the patients to address to an eye specialist. Also, the lack of data concerning functional aspects, because of unperformed visual fields at the initial consultation or follow-ups, in order to be able to compare. Moreover, studied parameters have not been assessed depending on the type of occlusion. 

## Conclusions

The findings of the current study fortify the correlation between glaucoma as a risk factor for retinal venous occlusion development, the intraocular pressure and optic nerve cupping as prediction factors in retinal venous occlusions, the association of a well-controlled preexisting glaucoma with no effect on the progression of the retinal venous occlusions and the development of a neovascular glaucoma with a much aggressive and different path of disease progression and also an aspect of prediction. Moreover, we hope that with a new technology that we are looking for in the future we will be able to quantify all these aspects and others in a much better and detailed manner.

**Conflict of Interest statement**

The authors state no conflict of interest.

**Informed Consent and Human and Animal Rights statement**

Informed consent has been obtained from all individuals included in this study.

**Authorization for the use of human subjects**

Ethical approval: The research related to human use complies with all the relevant national regulations, institutional policies, is in accordance with the tenets of the Helsinki Declaration, and has been approved by the Ethics Committee of “Victor Babeş” University of Medicine and Pharmacy, Timişoara, Romania.

**Acknowledgements**

None.

**Sources of Funding**

None.

**Disclosures**

None.
